# Meta-review of implementation determinants for policies promoting healthy diet and physically active lifestyle: application of the Consolidated Framework for Implementation Research

**DOI:** 10.1186/s13012-021-01176-2

**Published:** 2022-01-06

**Authors:** Karolina Lobczowska, Anna Banik, Katarzyna Brukalo, Sarah Forberger, Thomas Kubiak, Piotr Romaniuk, Marie Scheidmeir, Daniel A. Scheller, Juergen M. Steinacker, Janine Wendt, Katarzyna Wieczorowska-Tobis, Marleen P. M. Bekker, Hajo Zeeb, Aleksandra Luszczynska

**Affiliations:** 1 Department of Psychology in Wroclaw, SWPS University of Social Sciences and Humanities, Ostrowskiego Street 30b, PL53238 Wroclaw, Poland; 2 Department of Health Policy, School of Health Sciences in Bytom, Medical University of Silesia in Katowice, 18 Piekarska Street, PL41902 Bytom, Poland; 3 Leibniz Institute for Prevention Research and Epidemiology – BIPS, Achter Street 30, D28359 Bremen, Germany; 4 Johannes Gutenberg University Mainz, Institute of Psychology, Binger Street 14-16, D55122 Mainz, Germany; 5 Department of Internal Medicine, Division of Sports and Rehabilitation Medicine, University Hospital Ulm, Leimgrubenweg 14, D89075 Ulm, Germany; 6 Department of Palliative Medicine, Poznan University of Medical Sciences, Russa Street 55, PL61245 Poznan, Poland; 7 Wageningen University and Research, Health and Society Group, Center for Space, Place and Society, P.O. Box 8130, Bode 60, 6700 EW Wageningen, the Netherlands; 8 Melbourne Centre for Behavior Change, Melbourne School of Psychological Sciences, University of Melbourne, Redmond Barry Building, Parkville Campus, Melbourne, VIC 3010 Australia

**Keywords:** Policy, Implementation, Diet, Physical activity, Sedentary behavior, Barrier, Facilitator, Consolidated Framework For Implementation Research

## Abstract

**Background:**

Although multiple systematic reviews indicate that various determinants (barriers and facilitators) occur in the implementation processes of policies promoting healthy diet, physical activity (PA), and sedentary behavior (SB) reduction, the overarching synthesis of such reviews is missing. Applying the Consolidated Framework for Implementation Research (CFIR), this meta-review aims to (1) identify determinants that were systematically indicated as occurring during the implementation processes and (2) identify differences in the presence of determinants across reviews versus stakeholder documents on healthy diet/PA/SB policies, reviews/stakeholder documents addressing healthy diet policies versus PA/SB policies targeting any population/setting, and healthy diet/PA/SB policies focusing on school settings.

**Methods:**

A meta-review of published systematic scoping or realist reviews (*k* = 25) and stakeholder documents (*k* = 17) was conducted. Data from nine bibliographic databases and documentation of nine major stakeholders were systematically searched. Included reviews (72%) and stakeholder documents (100%) provided qualitative synthesis of original research on implementation determinants of policies promoting healthy diet or PA or SB reduction, and 28% of reviews provided some quantitative synthesis. Determinants were considered strongly supported if they were indicated by ≥ 60.0% of included reviews/stakeholder documents.

**Results:**

Across the 26 CFIR-based implementation determinants, seven were supported by 66.7–76.2% of reviews/stakeholder documents. These determinants were cost, networking with other organizations/communities, external policies, structural characteristics of the setting, implementation climate, readiness for implementation, and knowledge/beliefs of involved individuals. Most frequently, published reviews provided support for inner setting and individual determinants, whereas stakeholder documents supported outer and inner setting implementation determinants. Comparisons between policies promoting healthy diet with PA/SB policies revealed shared support for only three implementation determinants: cost, implementation climate, and knowledge/beliefs. In the case of healthy diet/PA/SB policies targeting school settings, 14 out of 26 implementation determinants were strongly supported.

**Conclusions:**

The strongly supported (i.e., systematically indicated) determinants may guide policymakers and researchers who need to prioritize potential implementation determinants when planning and monitoring the implementation of respective policies. Future research should quantitatively assess the importance or role of determinants and test investigate associations between determinants and progress of implementation processes.

**Trial registration:**

PROSPERO, #CRD42019133341

**Supplementary Information:**

The online version contains supplementary material available at 10.1186/s13012-021-01176-2.

Contributions to the literature

- Using the Consolidated Framework for Implementation Research, this study provides an overarching synthesis of evidence accumulated in reviews and stakeholder documents, reporting the occurrence of barriers and facilitators of implementation of policies targeting healthy diet, physical activity, or sedentary behavior.
- Seven determinants were indicated as occurring in implementation processes in 66.7–76.2% of analyzed reviews/stakeholder documents. These were: cost, networking with other organizations/communities, external policies, structural characteristics of the setting, implementation climate, readiness for implementation, and knowledge/beliefs.
- The findings may inform policymakers, implementers, and researchers in preselecting or narrowing down the number of potential implementation determinants when planning and monitoring implementation.

## Introduction

According to the Global Burden of Disease Study [1], the number of deaths attributable to poor diet (e.g., low fruit and vegetable intake, high energy-dense food intake) and low levels of physical activity (PA) has significantly increased between 2007 and 2017. Poor diet and low levels of physical activity are key risk factors for noncommunicable diseases, such as cardiovascular diseases, cancer, type-2 diabetes [1], and obesity [2]. The number of public policies directly or indirectly aiming at changes in dietary and physical activity behaviors has been growing in the last decade. The World Cancer Research Fund [3] identified over 690 national-level nutrition policies and over 150 national-level physical activity-promoting policies.

Policies may be defined as actions that are developed and implemented to directly or indirectly achieve specific goals within a society, such as better health through changes in dietary behaviors, PA promotion, and a reduction of sedentary behavior (SB) [4, 5]. National and regional governments participate in policy development and implementation. In contrast, interventions may be defined as actions with similar goals but not yet endorsed, enabled, or executed by regional or national governments or supranational organizations that have legal powers [5]. In contrast to interventions, policies result from value-driven decision-making processes in a setting where multiple values and interests are negotiated toward a shared consensus [6]. Thus, policies are evidence-based only to a limited extent [6].

Policy implementation may be defined as the process of putting to use or integrating a policy within a setting or a system, or the process of maintaining the use and capacity of a policy [7]. Implementation is a two-way social process through which policies are operationalized within an organization/community or a process in which practices are operationalized into policies [8]. The process involves implementation actors, implementation settings, implementation strategies, target population, and characteristics of a policy (e.g., its content) interacting with the broader cultural, social, economic, and political context [8, 9]. The characteristics of the context, setting, implementation actors, and target populations may constitute implementation determinants (or implementation conditions) that occur during the implementation [10]. Barriers (impediments) and facilitators (enablers) may also refer to the characteristics of the strategies used to influence the implementation [10].

### Key implementation determinants: existing evidence and its limitations

Systematic reviews have already discussed which barriers and facilitators occur during the implementation of policies and interventions to promote healthy diet, an increase in PA, and SB reduction [11–15]. Some reviews are narrowly focused and identify implementation determinants for policies with specific aims or settings (e.g., taxation for sugar-sweetened beverages) [16, 17]. Others have a moderately broad focus (e.g., healthy diet policies for any population in any setting) [18], or they attempt to synthesize evidence for the presence of determinants in the implementation of policies targeting various health behaviors across populations and settings (e.g., any obesity-related policies operating in any setting) [19]. Existing meta-reviews provide overviews of the occurrence of implementation determinants, assuming that the same determinants operate during the implementation of policies vs. interventions and that the same determinants are operating in case of the implementation of policies promoting healthy diet vs physically active lifestyle [19–21]. However, the comparisons of determinants (e.g., their relevance for healthy diet vs. PA/SB policies) were not conducted; thus, the assumption of common implementation determinants should be investigated further [20, 21].

Meta-reviews by Horodyska et al. [20, 21] discussed implementation processes, strategies, and determinants that were elicited in research that most frequently analyzed interventions (i.e., actions that do not involve national/regional governments). A synthesis of research that directly focus on the determinants of policy implementation processes is missing.

Childhood and adolescence are critical developmental periods when dietary and PA habits are formed, with schools representing the critical environment for the implementation of policies aimed at obesity prevention in young people [12–14, 22]. Research has provided evidence for numerous determinants operating during the implementation of either healthy diet policies [13] or PA/SB policies specific to the school setting [12, 14] as schools are the major implementers of healthy diet and PA/SB policies [22]. A synthesis of studies on key determinants that occur in the implementation of dietary, PA, and SB policies for the school setting is missing.

Using the accumulating evidence, major international and national stakeholders are issuing documents on developing, implementing, and evaluating healthy diet and PA/SB policies (e.g., the World Health Organization, the National Institute of Clinical Excellence). These documents are developed to guide governments in the formation and implementation of national and regional strategies and policies [22]. A synthesis of stakeholder documents may help to identify similarities/differences between empirical evidence (accumulating in reviews) and policy-guiding documents. It is unclear whether or how published reviews differ in their findings on implementation determinants, compared to the position of stakeholders, thus guiding the decisions of policymakers and practitioners.

### The Consolidated Framework for Implementation Research

Across the theoretical frameworks describing implementation determinants, the Consolidated Framework for Implementation Research (CFIR) [23] is among the most frequently used by implementation researchers and practitioners [24]. The CFIR [23] is a descriptive framework that lists 26 key determinants of implementation grouped into five broad domains: (1) characteristics of policies that may determine implementation (e.g., complexity); (2) the outer setting characteristics (e.g., networking with other organizations); (3) the inner setting characteristics (e.g., organizational climate); (4) individual-level determinants (e.g., knowledge and beliefs); and (5) characteristics of implementation processes (e.g., implementation plans). The CFIR merely lists the 26 determinants that may occur during the implementation processes. As a descriptive framework, the CFIR does not provide insight into how the determinants operate (e.g., which stages of implementation they hinder or facilitate; whether or how they are linked with other characteristics of the implementation processes or implementation outcomes).

Existing meta-reviews addressing aspects of the implementation of policies and interventions promoting healthy diet and a physically active lifestyle described best practices and determinants without referring to a specific implementation framework [20] or using generic frameworks such as Reach-Efficacy-Adoption-Implementation-Maintenance (RE-AIM) [21]. Although RE-AIM is popular among implementation researchers [24], it does not provide a comprehensive list of specific implementation determinants. Research on the criteria for the selection of the framework indicated that optimal frameworks are characterized by high usability (e.g., inclusion of relevant constructs; the use of the framework in research and practice), testability (e.g., the scale to which the framework contributes to evidence accumulation), and acceptability (e.g., being familiar to key stakeholders, including researchers and practitioners) [25]. The CFIR proposes well-defined implementation determinants and has been extensively used in research that specifically aimed to elicit the key determinants of implementation processes in various settings and across numerous target populations [26–28]. Therefore, the CFIR was selected as the guiding framework for this review.

### Aims

Using the CFIR framework and methods of meta-synthesis of reviews and stakeholder documents, the present study identifies which determinants occur in the processes of implementation of policies targeting healthy diet, PA promotion, and/or SB reduction. In particular, we intended to specify the following: (1) Which determinants from five domains of the CFIR [23] were identified as occurring in the policy implementation process? (2) What were the differences between determinants identified in reviews compared to those identified in stakeholder documents? (3) What were the differences between determinants of policy implementation that were identified in reviews/stakeholder documents addressing healthy diet policies only and those that were identified in reviews/stakeholder documents addressing PA/SB policies? (4) What were the determinants of healthy diet and PA/SB policy implementation identified in reviews/documents addressing a specific setting, i.e., schools?

We also explored whether the evidence accumulated in reviews/stakeholder documents would allow for synthesizing the roles played by the CFIR-based determinants, namely associations between determinants and other constructs operating during implementation processes, as well as the direction of these associations (hindering or facilitating the progress of implementation processes).

## Method

### Materials and general procedures

A meta-review (systematic review of reviews) [29]), integrating empirical evidence from existing reviews and stakeholder documents was conducted. This study was conducted in accordance with the Preferred Reporting Items for Systematic Reviews and Meta-Analyses (PRISMA) guidelines [30, 31]. It was registered with the PROSPERO database (no. CRD42019133341). Two types of documents were retrieved and analyzed: (1) peer-reviewed systematic reviews [32], or scoping [33], or realist reviews [34], analyzing original studies (henceforth: reviews), and (2) documents issued by major international stakeholders (henceforth: stakeholder documents).

### Published reviews: search strategy and criteria of inclusion and exclusion

The following databases were searched: MEDLINE, Academic Search Ultimate, AGRICOLA, PsycINFO, PsycARTICLES, Health Source: Nursing/Academic Edition, the Cochrane Database of Systematic Reviews (CDSR), Database of Abstracts of Reviews of Effects (DARE), and Scopus. Documents published between the inception of the databases and February 2020 were included. Additionally, manual searches of reference lists of reviews were conducted, and keyword-based searches of implementation journals (e.g., *Implementation Science*, *Health Research Policy and Systems*, *Policy Studies*) were performed. In line with the search strategies used in previous meta-reviews [13, 20, 21, 35], combinations of five groups of keywords were applied, referring to: (1) implementation; (2) barriers and facilitators; (3) the type of action (e.g., policy); (4) the design of the study (e.g., systematic review); and (5) the behavioral outcomes (e.g., physical activity; for details, see Supplement 2, Table S1).

**Fig. 1:**
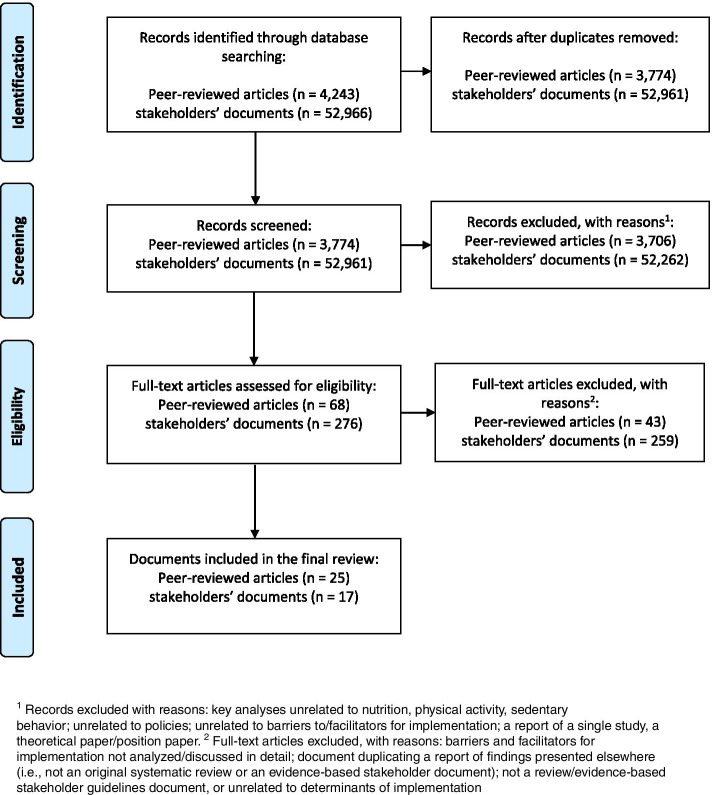
The flow chart: selection processes for peer-reviewed articles and stakeholder documents

The stages of the data selection process are presented in Fig. 1. The initial search step yielded *k* = 4243 records, which used a combination of keywords from all five categories in the title, abstract, or subject terms. Each abstract was screened by two researchers (KL and AB), and any potential conflicts were resolved through discussion with a third researcher (AL).

We included quantitative and qualitative reviews, applying methods of systematic reviews [32], scoping reviews [33], or realist reviews [34]. The reviews were included if they (1) were published in peer-reviewed English-language journals and (2) provided an analysis of original research on implementation determinants for policies promoting healthy diet or PA or SB reduction. The following types of documents were excluded: dissertations, protocols, conference materials, book chapters, reviews that did not test the role/effects of implementation determinants, publications addressing policies targeting other health behaviors (e.g., smoking), reviews of policy guidelines (not reviews of original research), reports of one original study or testing determinants in one implementation process, and reviews of theoretical models.

### Stakeholder documents: search strategy and criteria of inclusion and exclusion

In line with previous research [20, 21], we included documents from stakeholders representing governmental and non-governmental organizations, issuing evidence-based policy guidelines (in English) for diet, PA, and/or SB policies at the national or international level. The documents from the following stakeholders were included: the European Commission, the World Health Organization, the National Institute for Health and Care Excellence (United Kingdom), Centers for Disease Control and Prevention (USA), National Academy of Medicine (USA), Australian Department of Health, the National Health and Medical Research Council, the Organization for Economic Co-operation and Development, and the Food and Agriculture Organization of the United Nations.

Publicly available stakeholder websites (e.g., repositories of strategy documents, policy guidelines, and best practice guidelines) were searched to identify potentially relevant documents. The search was conducted from the inception of the databases until February 2020, using the same combination of five groups of keywords as those applied in the search for reviews (for details see, Supplement 2, Table S1). In line with previous research [20, 21, 36], the criteria for selecting the stakeholder documents were as follows: issued in English; discussing the stakeholder’s proposal of practice guidelines or best practices in policy development and/or implementation; addressing diet, PA, and/or SB policies; using research evidence to discuss implementation process and/or its determinants (e.g., including references to original research or reviews of original research).

The exclusion criteria applied in the screening of stakeholder documents were the same as those used for reviews. The initial search identified *k* = 57, 209 potentially relevant documents (Fig. 1), each of which was screened by at least two researchers (PR, KB, KWT, MS, DAS, JW, and KL or AL).

### Data extraction

All stages of data extraction, selection, and coding were conducted by at least two researchers. Any disagreements during the data extraction process were resolved by a consensus involving a third researcher [32]. Descriptive data (see Supplement 1) were extracted by two researchers (KL and AL) and verified by a third researcher (AB). Extracted data included: (1) the descriptive characteristics of the included reviews/stakeholder documents (the number, design, and objectives of studies included in the review; the framework used to guide or organize the findings of the review/document; target population and settings; analyzed behavior; the type of action [intervention and/or policies]); (2) information about implementation determinants (the name, definition, and operationalization of determinants, as provided by authors of original documents); (3) the type of support provided for the respective determinant, as reported in the results sections of the included reviews; and (4) data necessary for the quality evaluation of reviews/stakeholder documents.

### Data coding

Supplement 2 (Table S3) provides definitions and criteria applied in coding the following constructs: policy, implementation, healthy diet policy, PA/SB policy, and school setting policies (addressing healthy diet, PA/SB).

The coding for the CFIR determinants was conducted using the descriptions and definitions included in Damschroder et al. [23]. All retrieved barriers and facilitators, derived separately from reviews and stakeholder documents, were allocated into five domains of the CFIR [23]. The domains of the CFIR include 26 implementation determinant categories (*k*): (1) policy characteristics (*k* = 8; policy source, evidence strength, relative advantage, adaptability, triability, complexity, quality, cost); (2) outer setting characteristics (*k* = 4; target group needs and resources, networking with other organizations, peer pressure, external policies); (3) inner setting characteristics (*k* = 5; structural character, networks and communication, culture/norms/values, implementation climate, readiness for implementation); (4) characteristics of individuals (*k* = 5; knowledge/beliefs, self-efficacy, stages of change/enthusiasm, identification with organization, motivation/values/capacity); and (5) implementation process (*k* = 4; planning, engaging leaders/agents/champions, executing plans, reflecting and evaluating). The researchers (KL, AB, PR, KB, MS, KWT, DAS, and JW) worked in pairs independently to extract and code the data, and any disagreements were discussed with a third researcher (AL). Further details regarding coding implementation determinants using 26 CFIR [23] categories are reported in Supplement 2, Table S3.

### Risk of bias assessment

The risk of bias in reviews was assessed using phases two and three of the ROBIS tool [37]. The risk of bias in stakeholder documents was assessed using the Methodological Quality Checklist for Stakeholder Documents and Position Papers, (MQC-SP) [20, 21]. Two researchers (KL and AB) independently rated all included reviews and stakeholder documents, and disagreements were resolved by involving a third researcher (AL). The obtained scores are reported in Supplement 1. For the two types of analyzed documents, the concordance coefficients (intra-class correlation) for quality assessment ranged from .71 to .90 (all *p*s < .003).

### Data analysis and synthesis

Reviews and stakeholder documents were coded as not corroborating (-) or providing corroboration (+) for the presence of the determinant in the process of policy implementation (Supplement 2, Tables S4 and S5). The reviews of original quantitative studies were coded as providing corroboration for the presence of the respective determinant in implementation processes if the results section of the review indicated that: (1) the respective determinant was identified in the original studies as being significantly associated with another characteristic of the implementation process or its outcome (e.g., acceptability of a policy); (2) the determinant was identified in the original studies as occurring during the implementation process (e.g., the level of intensity/frequency or median/range values of the determinant that was assessed through a questionnaire and interpreted as indicating the presence of the determinant in processes analyzed in a respective study). The included reviews used various thresholds for identifying the occurrence of a determinant (e.g., mean/range provided in at least one study or at least 50% of participants in the original study mentioning that the determinant influenced the implementation). Therefore, in the present review, the determinant was coded as “indicated in the original review” if its results section concluded that the determinant was present in the respective implementation process. The reviews of qualitative studies were coded as providing corroboration for the respective determinant if the results section of the review indicated that the respective determinant was identified in original qualitative data (e.g., the thematic analysis indicated that participants recognized a respective factor as influencing implementation processes).

Stakeholder documents were coded as providing corroboration for the presence of the respective determinant in the implementation process if the sections of the documents discussing guidelines/best practices listed a determinant and indicated its significance/importance/need for consideration in the process of policy implementation, as well as providing reference to original research backing a respective statement.

In line with previous meta-reviews synthesizing evidence for healthy diet or PA [38, 39], support thresholds of 50.0% and 60.0% were applied. Determinants that were indicated in between 50.0 and 59.9% of reviews/stakeholder documents were considered obtaining preliminary support for their presence in the implementation process. Determinants that were indicated in ≥ 60.0% of analyzed reviews/stakeholder documents were considered obtaining strong support for the presence of a determinant in the implementation processes.

Additionally, to synthesize the findings on the role of the determinants, we coded if the methods of the included reviews indicated that determinants were categorized (based on evidence obtained in original studies) as barriers or facilitators. This type of coding was conducted in the reviews only. Reviews were coded for the provision of evidence for the significant association between a respective determinant and any other implementation process variables (or implementation outcomes or effectiveness of policies). Finally, the reviews were coded in terms of conducting quantitative analysis, showing the proportion/frequency of the occurrence of a determinant in original research (compared to a narrative synthesis only, illustrating the occurrence of the determinants with examples of references).

Comparisons of reviews vs. stakeholder documents and healthy diet vs. PA/SB policies were conducted. We listed determinants that obtained strong support, both overlapping and differing according to document type and policy type. Comparisons of diet vs. PA/SB policies were limited to reviews/stakeholder documents that addressed only the implementation of policies targeting the respective behavior (i.e., healthy diet policies only vs. PA/SB policies only). Data referring to healthy diet and PA/SB policies in the school setting were summarized, listing the implementation determinants that obtained strong support for their presence in the implementation process.

## Results

### Description of analyzed material

The final selection included 25 reviews [11–13, 16–19, 40–57] and 17 stakeholder documents [22, 58–73]. The characteristics of target populations, behaviors targeted by policies, and policy settings are reported in Supplement 2, Table S2.

The scores obtained using ROBIS [37] and MCQ-SP [20, 21], indicating the quality of the included reviews and documents, are reported in Supplement 1. Regarding the risk of bias, 48% (*k* = 12) of reviews evaluated as representing a low risk of bias across five criteria of ROBIS [37], 24% (*k* = 6) had a low risk across four criteria, and 8% (*k* = 2) had a low risk in three criteria. The remaining 20% (*k* = 5) of the reviews were evaluated as having a high or unclear risk in ≥ 3 criteria. Among the stakeholder documents, 47% (*k* = 8) had a low risk of bias (high quality in MQC-SP tool) [20, 21], 29% (*k* = 5) had moderate quality, and 24% had a high risk/low quality (*k* = 4).

Across reviews, 20.0% (5 out of 25) [41, 44, 52, 53, 56] included quantitative studies only, and the remaining 20 reviews combined data obtained from quantitative and qualitative studies (Supplement 1). Fifty-six percent of the reviews (14 out of 25) specified their aims to identify facilitators and barriers (determinants positively or negatively associated with the implementation process or its outcomes). However, only 3 of the 25 reviews [13, 50, 56] reported any quantitative results that indicated associations between a determinant and any other implementation process-related variable (Supplement 1). Only one review reported findings of a meta-analysis showing non-significant weighted effects of determinants (facilitators) on implementation outcome variables, based on three included studies [56] (Supplement 1).

All reviews (25 out of 25) provided a narrative summary of original qualitative and/or quantitative research, listing the determinants that occurred in the implementation process (Supplement 1). The majority of reviews (72.0%, 18 out of 25) provided a narrative synthesis only, in which a determinant identified in original studies was indicated, followed by examples of original studies that reported respective determinants. Only 7 of the 25 reviews (28.0%) [12, 17, 40, 48, 50, 51, 56] provided a frequency analysis, clarifying a proportion of original studies indicating the occurrence of a respective determinant, compared to the total number of relevant original studies.

### Support for the occurrence of CFIR-based determinants

**Fig. 2:**
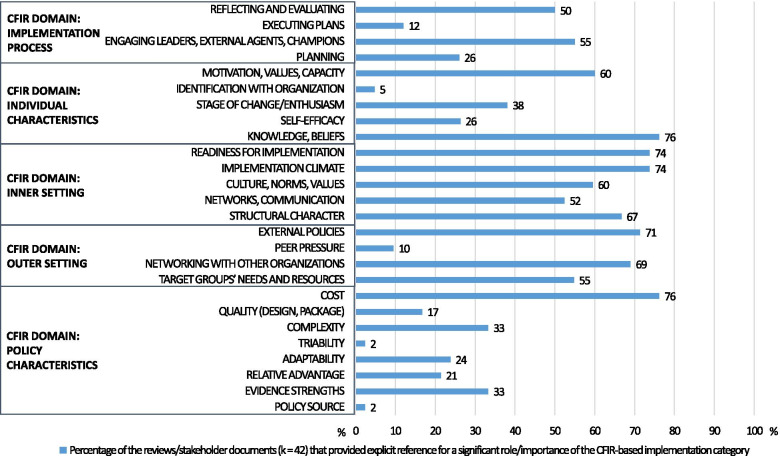
The support for policy implementation determinants according to the CFIR, obtained in *k* = 42 reviews and stakeholder documents

Across all reviews and stakeholder documents included in this study (*k* = 42), 7 of the 26 CFIR determinants received strong support (see Table 1, Fig. 2). In particular, strong support (76.2%, 32 out of 42 reviews/documents) was obtained for cost, the only determinant from the policy characteristic domain. Regarding the outer setting domain, two determinants received strong support, namely networking between organizations/institutions/communities (69.0%, 29 out of 42 reviews/documents) and external policies (71.4%, 30 out of 42 reviews/documents). Three inner-setting determinants received strong support: implementation climate (73.8%, 31 out of 42 reviews/documents), readiness for implementation (73.8%, 31 out of 42 reviews/documents), and structural determinants (66.7%, 28 out of 42 reviews/documents). One individual characteristic domain determinant obtained strong support (knowledge/beliefs; 76.2%, 32 out of 42 reviews/documents), whereas no process-related determinants were strongly supported.

**Table 1:** Percentage of systematic reviews and stakeholder documents corroborating occurrence of CFIR-based implementation determinants

Policy implementation determinants: CFIR domains and categories of determinants	Total: % (number of reviews/documents supporting the determinant in all ***k*** = 42 reviews/documents)	Reviews vs. stakeholder documents comparisons		Diet vs. PA/SB comparisons (reviews and stakeholder documents)		Diet, PA, SB policies in schools: % (number of reviews/documents supporting the determinant in ***k*** = 10 reviews/documents)
		Reviews: % (number of reviews supporting the determinant in ***k*** = 25 reviews)	Stakeholder documents: % (number of documents supporting the determinant in ***k*** = 17 documents)	Diet: % (number of reviews/documents supporting the determinant in ***k*** = 12 reviews/documents)	PA/SB: % (number of reviews/documents supporting the determinant in ***k*** = 9 reviews/documents)	
**Domain: Policy characteristic**						
Intervention source	2 % (1 out of 42)	4 % (1 out of 25)	0	0	0	0
Evidence strengths	33 % (14 out of 42)	24 % (6 out of 25)	47 % (8 out of 17)	42 % (5 out of 12)	0	0
Relative advantage	21 % (9 out of 42)	28 % (7 out of 25)	12 % (2 out of 17)	8 % (1 out of 12)	22 % (2 out of 9)	30 % (3 out of 10)
Adaptability	24 % (10 out of 42)	24 % (6 out of 25)	24 % (4 out of 17)	8 % (1 out of 12)	11 % (1 out of 9)	20 % (2 out of 10)
Triability	2 % (1 out of 42)	4 % (1 out of 25)	0	0	0	0
Complexity	33 % (14 out of 42)	*56 % (14* out of *25)*	0	33 % (4 out of 12)	**78 % (7 out of 9)**	**70 % (7 out of 10)**
Quality (design, package)	17 % (7 out of 42)	28 % (7 out of 25)	0	25 % (3 out of 12)	11 % (1 out of 9)	30 % (3 out of 10)
Cost	**76 % (32** out of **43)**	**76 % (19** out of **25)**	**77 % (13** out of **17)**	**83 % (10** out of **12)**	**67 % (6 out of 9)**	**70 % (7 out of 10)**
Mean % across determinants from policy characteristic domain	26 %	30 %	20 %	25 %	24 %	28 %
**Domain: Outer setting**						
Target groups’ needs and resources	*55 % (23 out of 42)*	48 % (12 out of 25)	**65 % (11 out of 17)**	33 % (4 out of 12)	44 % (4 out of 9)	**60 % (6 out of 10)**
Networking with other organizations	**69 % (29 out of 42)**	**64 % (16 out of 25)**	**77 % (13 out of 17)**	**75 % (9 out of 12)**	*56 % (5 out of 9)*	**60 % (6 out of 10)**
Peer pressure	10 % (4 out of 42)	16 % (4 out of 25)	0	17 % (2 out of 12)	11 % (1 out of 9)	0
External policies	**71 % (30 out of 42)**	**64 % (16 out of 25)**	**82 % (14 out of 17)**	**83 % (10 out of 12)**	*56 % (5 out of 9)*	**60 % (6 out of 10)**
Mean % across determinants from outer setting domain	*51 %*	48 %	*56 %*	*52 %*	42 %	45 %
**Domain: Inner setting**						
Structural character	**67 % (28 out of 42)**	**68 % (17 out of 25)**	**65 % (11 out of 17)**	*58 % (7 out of 12)*	**78 % (7 out of 9)**	**90 % (9 out of 10)**
Networks, communication	*52 % (22 out of 42)*	40 % (10 out of 25)	**71 % (12 out of 17)**	*50 % (6 out of 12)*	44 % (4 out of 9)	40 % (4 out of 10)
Culture, norms, values	60 %* (25 out of 42)	**64 % (16 out of 25)**	*53 % (9 out of 17)*	*58 % (7 out of 12)*	33 % (3 out of 9)	**60 % (6 out of 10)**
Implementation climate	**74 % (31 out of 42)**	**80 % (20 out of 25)**	**65 % (11 out of 17)**	**67 % (8 out of 12)**	**78 % (7 out of 9)**	**80 % (8 out of 10)**
Readiness for implementation	**74 % (31 out of 42)**	**72 % (18 out of 25)**	**77 % (13 out of 17)**	**67 % (8 out of 12)**	44 % (4 out of 9)	**80 % (8 out of 10)**
Mean % across determinants from inner setting domain	**65 %**	**65 %**	**66 %**	**60 %**	*56 %*	**70 %**
**Domain: Individual characteristics**						
Knowledge, beliefs	**76 % (32 out of 42)**	**84 % (21 out of 25)**	**65 % (11 out of 17)**	**83 % (10 out of 12)**	**89 % (8 out of 9)**	**90 % (9 out of 10)**
Self-efficacy	26 % (11 out of 42)	44 % (11 out of 25)	0	17 % (2 out of 12)	33 % (3 out of 9)	**70 % (7 out of 10)**
Stage of change/enthusiasm	38 % (16 out of 42)	*56 % (14 out of 25)*	12 % (2 out of 17)	33 % (4 out of 12)	**67 % (6 out of 9)**	**70 % (7 out of 10)**
Identification with organization	5 % (2 out of 42)	8 % (2 out of 25)	0	8 % (1 out of 12)	0	0
Motivation, values, capacity	60 %* *(25 out of 42)*	**72 % (18 out of 25)**	41 % (7 out of 17)	42 % (5 out of 12)	**78 % (7 out of 9)**	**70 % (7 out of 10)**
Mean % across determinants from individual characteristics domain	41 %	*53 %*	24 %	37 %	*53 %*	**60 %**
**Domain: Implementation process**						
Planning	26 % (11 out of 42)	24 % (6 out of 25)	29 % (5 out of 17)	17 % (2 out of 12)	33 % (3 out of 9)	10 % (1 out of 10)
Engaging leaders, external agents, champions	*55 % (23 out of 42)*	**64 % (16 out of 25)**	41 % (7 out of 17)	42 % (5 out of 12)	*56 % (5 out of 9)*	**80 % (8 out of 10)**
Executing plans	12 % (5 out of 42)	16 % (4 out of 25)	6 % (1 out of 17)	8 % (1 out of 12)	11 % (1 out of 9)	10 % (1 out of 10)
Reflecting and evaluating	*50 % (21 out of 42)*	40 % (10 out of 25)	**65 % (11 out of 17)**	42 % (5 out of 12)	44 % (4 out of 9)	40 % (4 out of 10)
Mean % across determinants from implementation process domain	36 %	36 %	35 %	27 %	36 %	35 %

Note: *PA* physical activity, *SB* sedentary behavior, *CFIR* Consolidated Framework for Implementation Research. %—the percentage of the reviews/stakeholder documents that reported the occurrence of respective CFIR-based domain/category of implementation determinants. Total—reviews/stakeholder documents (*k* = 42) addressing implementation determinants for healthy diet, PA/SB policies. Reviews—reviews (*k* = 25) addressing implementation determinants for healthy diet, PA/SB policies. Stakeholder—stakeholder documents (*k* = 17) addressing implementation determinants for healthy diet, PA/SB policies. Diet—reviews/stakeholder documents (*k* = 12) addressing implementation of healthy diet policies across various populations/settings. PA/SB—reviews/stakeholder documents (*k* = 9) addressing implementation of PA/SB policies across various populations/settings. Schools—reviews/documents (*k* = 10) addressing implementation of healthy diet or PA/SB policies for school settings. The percentages of implementation determinants that were corroborated in ≥ 50.0% up to 59.9% of reviews/stakeholder documents (considered preliminarily supported) are italicized. The percentages of implementation determinants corroborated in ≥ 60% of analyzed reviews/stakeholder documents (considered strongly supported) are in bold font. *in the case where between 55.5 and 59.99% of reviews/stakeholder documents supported the occurrence of a determinant, they were rounded to 60%; however, they were not considered an indication of strong support because the actual values were lower than 60.0%

### Support for the occurrence of CFIR-based determinants of implementation processes: reviews vs. stakeholder documents

Across published reviews, knowledge/beliefs (84.0%, 21 out of 25 reviews), implementation climate (80.0%, 20 out of 25 reviews), cost (76.0%, 19 out of 25 reviews) constituted the top three determinants (most frequently indicated), followed by readiness for implementation (72.0%, 18 out of 25 reviews), motivation/values/capacity (72.0%, 18 out of 25 reviews), and external policies (64.0%, 16 out of 25 reviews). Overall, 10 out of 26 implementation determinants were indicated in at least 60.0% of the reviews (Table 1 and Supplement 2, Table S4).

Compared to published reviews, the barriers/facilitators most frequently corroborated by stakeholder documents differed in terms of the top determinants (Table 1 and Supplement 2, Table S5). The most frequently corroborated characteristics included external policies (82.4%, 14 out of 17 documents) and, equally, networking with other organizations/institutions/communities, readiness for implementation, and cost (each characteristic was corroborated in 76.5% reviews/stakeholder documents, i.e., 13 out of 17 documents). The characteristics of networks/communication were corroborated in 70.6% (12 out of 17) of the stakeholder documents. Additionally, five determinants were corroborated equally often (in 64.7%, i.e., 11 out of 17 documents), namely target groups’ needs and resources, structural characteristics, implementation climate, knowledge/beliefs, and reflecting/evaluating. Overall, 10 implementation determinants obtained strong support in stakeholder documents (Table 1 and Supplement 2, Table S5).

### Support for the occurrence of CFIR-based determinants of implementation processes: healthy diet vs. physical activity/sedentary behavior policies

The comparison of healthy diet vs. PA/SB policies yielded several differences in policy implementation determinants that received strong support (Table 1 and Supplement 2, Tables S4 and S5). Regarding reviews/stakeholder documents (*k* = 12, including eight reviews and four stakeholder documents) addressing healthy diet policies, strong support was obtained for six implementation determinants, including: cost (83.3%, 10 out of 12 reviews/documents), knowledge/beliefs (83.3%, 10 out of 12 reviews/documents), and implementation climate in the inner setting (66.7%, 8 out of 12 reviews/documents). Strong support was also obtained for external policies (83.3%, 10 out of 12 reviews/documents), networking with organizations/institutions/communities in the outer setting (75.0%, 9 out of 12 reviews/documents), and readiness for implementation in the inner setting (66.7%, 8 out of 12 reviews/documents). The latter three determinants did not reach a strong support threshold in the case of PA/SB policies.

Regarding reviews and stakeholder documents addressing PA/SB policies only (*k* = 9, reviews/stakeholder documents), three determinants that received strong support were common with those indicated in reviews/documents addressing healthy diet policies, namely cost (66.7%, 6 out of 9 reviews/documents), implementation climate in the inner setting (77.8%, 7 out of 9 reviews/documents), and knowledge/beliefs (88.9%, 8 out of 9 reviews/documents). Additionally, four determinants received strong support in the case of PA/SB policies (but not in healthy diet policies), namely complexity within policy characteristic domain (77.8%, 7 out of 9 reviews/documents), structural characteristics of the inner setting (77.8%, 7 out of 9 reviews/documents), and two determinants from the domain of individual characteristics, in particular stages of change/enthusiasm (66.7%, 6 out of 9 reviews/documents), and motivation/values/capacity (77.8%, 7 out of 9 reviews/documents).

### Support for the occurrence of CFIR-based determinants of implementation processes: healthy diet and PA/SB policies in the school setting

Regarding the school setting, 10 reviews addressed the implementation of healthy diet and PA/SB policies solely in this setting (stakeholder documents did not address the school setting). Reviews provided strong support for three outer setting determinants, namely target group needs/resources, networking with other organizations, and external policies (all three supported in 60.0%, 6 out of 10 reviews). The presence of four inner setting determinants was strongly supported: structural characteristics (90.0%, 9 out of 10 reviews), implementation climate and implementation readiness (80.0% each, 8 out of 10 reviews), and institutional culture (60.0%, 6 out of 10 reviews). The occurrence of four individual characteristics was strongly supported: knowledge/beliefs (90.0%, 9 out of 10 reviews), self-efficacy, stages of change/enthusiasm, and motivation/values/capacity (70.0% each, 7 out of 10 reviews). Additionally, engaging leaders (80.0%, 8 out of 10 reviews) from the process domain was strongly supported, along with two determinants from the policy characteristics domain: complexity and cost (70.0% each, 7 out of 10 reviews). Overall, 14 out of the 26 categories of determinants were strongly supported (Table 1).

## Discussion

Applying the CFIR framework, this meta-review provides an overarching synthesis of evidence for the presence of determinants of implementation processes of policies aimed at healthy diet, promotion of physical activity, and a reduction of sedentary behaviors. Between 66.7% and 76.2% of analyzed reviews and stakeholder documents corroborated the occurrence of seven CFIR-based determinants in policy implementation processes. The determinants included cost, networking with other organizations/communities, external policies, structural characteristics of the setting, implementation climate, readiness for implementation, and knowledge/beliefs of the involved individuals.

Compared to stakeholder documents, the findings obtained in reviews show strong support for the occurrence of determinants from the individual characteristics domain (knowledge/beliefs, motivation/values/capacity). The difference may result, among others, from the use of frameworks such as the theoretical domain framework [74], focusing on individual characteristics of the target group and the implementers. Such frameworks were not used by stakeholders as the background to present the implementation determinants. Instead, stakeholders such as the World Health Organization often rely on their own frameworks [75], focus on policy development, and thus pinpoint outer setting determinants. The focus on outer setting implementation determinants in stakeholder documents becomes even more evident when analyzing the content of the barriers and facilitators listed in stakeholder documents (see Supplement 1) [22, 58, 76]. These outer setting determinants refer to inter-sectoral collaboration, co-occurring governmental regulations, national and local policies, characteristics of legal regulations, and funding schemes, operating in the respective country [22, 58, 76]. These implementation determinants are not well reflected in the CFIR, which accounts for the broader setting characteristics, but in a relatively general manner (e.g., accounting for “external policies”). In line with other approaches to policy implementation (e.g., the evidence-informed policy and practice framework) [77], so-called system-level determinants could be accounted for in a separate domain. These determinants may be divided into characteristics referring to other policies, economics (cf. [77]), or focus on macro-level determinants referring to legal contexts [8]. This conclusion is in line with a recent review on the use of the CFIR in original research, where a new CFIR domain of “characteristics of a system” was proposed [27].

In terms of strong support obtained for the occurrence of determinants of implementation processes, there are more differences than similarities when healthy diet policies are compared to PA/SB policies. Cost, implementation climate, and individuals’ knowledge/beliefs were commonly supported determinants. Implementation determinants strongly supported in reviews and documents analyzing PA/SB policies (but not healthy diet policies) included the complexity of policy implementation, structural characteristics of the setting, and enthusiasm of the individuals involved. Complexity (or rather, a lack thereof) and structural characteristics may play a role, particularly if the implementation takes place in specific settings (e.g., schools), where multiple complex policies are already operating; thus, simpler policies may be easier to integrate. Furthermore, the physical/built environment characteristics of such settings (e.g., a lack of stairs) may reduce the likelihood of successful implementation of PA policies [12, 43]. In contrast, reviews/stakeholder documents addressing the implementation of healthy diet policies strongly supported determinants such as networking with other organizations and readiness for implementation. The occurrence of determinants coded as “networking with other organizations” was reported in documents analyzing the implementation of food retail and food labeling policies (corroborating the importance of networking and communication between food producers, retailers, and customer organizations) [18, 40] or school-based policies (networking with parent organizations, food producers, food retail organizations) [42]. Conclusions should be drawn cautiously from the comparisons of implementation determinants for healthy diet and PA/SB policies, as the analyzed reviews/stakeholder documents were heterogeneous in terms of the type of policies and their breadth (e.g., healthy diet for preschoolers, food labeling, acceptability of taxation of sugar-sweetened beverages).

As many as 14 out of 26 implementation determinants were indicated in over 60% of published reviews analyzing determinants of implementation of school policies for healthy diet or PA/SB. One of the reasons for the large number of determinants occurring in schools’ implementation may be the characteristics of the policies themselves. For example, several analyzed policies dealt with complex education programs referring to healthy diet, consistent with changes in school food retail/catering, and/or integration of PA programs into complex curricula and limited built facilities [12, 13, 47]. Compared to taxation policies, the implementation of education policies may be dependent on multiple inner setting characteristics (e.g., built structures, training for implementers) or characteristics of individuals (teachers, students, parents, school managers). Consequently, eight out of ten determinants from these two CFIR domains were strongly supported. The inner setting domain and the individual characteristics domain are described in detail in the CFIR [23], in contrast to system-level complexities (e.g., legal solutions or strategic policy alignment) [27], which may be crucial, for example, for taxation policies.

Although the CFIR is among the most frequently used frameworks to capture implementation determinants [24, 28], and thus its application may facilitate cross-study comparisons, the framework has some limitations. First, it is a descriptive framework that lists the domains and included determinants [78], but does not provide an insight into the way the determinants may operate together (e.g., in a multidirectional manner, influencing each other and the implementation processes or implementation outcomes). In particular, if a complex system-logic approach is considered [79], the implementation of healthy diet and PA/SB policies should recognize the complex interplays (or associations) between as well as within the distinct domains included in the model (e.g., between inner setting and outer setting determinants). The data obtained in this meta-review provided no insight into the potential interplays between implementation determinants, mostly because neither the theoretical framework nor analyzed data included information on such interplays.

The CFIR was not developed with a focus on public policies but, rather, to address organizational or professional policies that do not have to comply with or are not set according to democratic requirements, consisting of the political deliberation and designation of social, collective problems, public values, and shared interests in the decision-making and formulation processes that are typical of public governmental policies [6]. Although the framework has been applied to public policy evaluations, it should be noted that the CFIR model offers a narrow, limited view on the characteristics mentioned above.

The meaning and effects of the seven most frequently occurring determinants across different policy types, contexts, and/or settings should be further explored by stakeholders, policymakers, and researchers. The specific context in which determinants operate (e.g., food retail or nutrition education) or directionality of their effects (facilitating or hindering the implementation) may differ across policies, depending on the target population, setting, and the content of policies. The associations between determinants and other factors operating in the implementation process or their influences on the progress of implementation or implementation outcomes remain unclear. The qualitative and quantitative data collected in the original research and the subsequently included reviews/stakeholder documents allow only to conclude which determinants are indicated as present and operating in the process of implementation of healthy diet, PA, or SB policies.

Regardless of these limitations, the seven implementation determinants supported in this meta-review may be considered the top priority when planning and monitoring the implementation of healthy diet and PA/SB policies. These determinants may be considered a safe choice in the research and practice of policy implementation if a preselection of implementation determinants is needed [28]. Policymakers, researchers, implementation actors, and other stakeholders should prepare strategies to address the respective determinants when planning for the implementation of healthy diet and PA/SB policies.

The present study has several limitations. The coding of the CFIR determinants relied on the specificity of the operationalization and descriptions of barriers and facilitators in reviews and stakeholder documents. Thus, several determinants were not assigned to any of the 26 implementation determinants (e.g., “putting daily physical activity in schedule”). Furthermore, several system-level determinants were not coded (e.g., “political climate promoting ‘being Australian’”) as they were not directly captured by the CFIR. Further theoretical developments are needed to better guide empirical research collecting evidence for the occurrence of determinants of policy implementation processes. Even the extended version of the CFIR [27] may not capture climate/geography-related barriers or specific system-level determinants. New hybrid frameworks that combine the CFIR with frameworks addressing the context of implementation processes [8] or clarifying the associations between determinants and other implementation-related constructs [80] may offer a better fit between the guiding framework and empirical data. Furthermore, the included reviews and stakeholder documents are highly heterogeneous in terms of the scope of the analyzed policies, target groups, policy settings, or quality of the review/stakeholder document. Due to the heterogeneity of the analyzed material and the variability regarding the risk of bias, conclusions, if any, should be drawn with great care. Although the heterogeneity of the aims and scope of included documents reduces the overlap in original studies (included in reviews), it may not be totally eliminated [29], and its effect should be investigated systematically. The CFIR [23] does not allow for a differentiation between the determinants referring to the target group, implementation support system actors (e.g., system administrators), or those who are directly responsible for implementation (e.g., educators). Therefore, the present study does not distinguish between determinants, such as the beliefs of the target population (e.g., children) and the beliefs of the implementers (e.g., teachers). Original quantitative research and reviews applied various theoretical frameworks and questionnaires, which may result in an increased likelihood of reporting some determinants, while missing others. The included reviews and stakeholder documents varied in terms of their focus on policies (the majority accounted for both policies and interventions). It is possible that the implementation of policies depends more on determinants from the outer setting (or the system level), whereas interventions may depend more on the inner setting determinants of individual characteristics.

The key limitation of the present study is the applied methods of analysis and synthesis. The obtained data allowed only counting the occurrence (compared to a lack of the presence of a respective determinant in the analyzed material). The majority (72%) of reviews lacked a quantitative analysis, for example, indicating the proportion of studies supporting vs. not supporting the occurrence of a respective determinant. The fact that a determinant was supported in a respective stakeholder document (vs. a lack of such support) may, among others, result from the influences of political context variables (e.g., political strategies and priorities of governments) [5, 6]. Future original research and reviews should provide quantitative data and quantitative analyses that would allow better estimation of the importance of a respective determinant.

## Conclusions

Despite these limitations, this study provides the first overarching synthesis of evidence accumulated in reviews and stakeholder documents on determinants facilitating and/or hindering the implementation of policies targeting healthy diet, an increase in PA, or SB reduction. The findings indicate seven determinants that are likely to occur in the implementation process, namely cost of implementation, networking with other organizations/communities, external policies, structural characteristics of the setting, implementation climate, readiness for implementation, and knowledge/beliefs of the involved individuals. The findings may inform policymakers, implementers, and researchers if they need to preselect or narrow down the number of potential implementation determinants when planning and monitoring the implementation of policies promoting healthy diet and physically active lifestyle.

## Supplementary Information

**Additional file 1: Supplement 1**: Details of data extraction and quality evaluation scores.

**Additional file 2: Supplement 2**: Details of searching strategy, analyzed materials, and data coding.

## Data Availability

The datasets used and analyzed during the current study are available from the corresponding author on a request.

## Abbreviations

CFIR: Consolidated Framework For Implementation Research
PA: Physical activity
SB: Sedentary behaviors
TDF: Theoretical domain framework
