# Supplementary material for: Meta-review of implementation determinants for policies promoting healthy diet and physically active lifestyle: application of the Consolidated Framework for Implementation Research
Source: Implement Sci. 2022 Jan 6;17:2. doi: 10.1186/s13012-021-01176-2 (PMC8734337; doi:10.1186/s13012-021-01176-2)
Supplement: Supplementary file 2 — Additional file 2: Supplement 2: Details of searching strategy, analyzed materials, and data coding. [file 13012_2021_1176_MOESM2_ESM.docx]

**Meta-review of implementation determinants for policies promoting healthy diet and physically active lifestyle: Application of the Consolidated Framework for Implementation Research**

**Supplement 2**

Supplementary Table S1

Full list of 5 groups of keywords applied in the searching strategy

| 1. **Implementation: implement*** |
| --- |
| 1. **Determinants, facilitators, and barriers:** barrier* OR facilitat* OR determinant* OR factor* OR affect* OR influenc* OR enhanc* OR improv* OR support* OR gap |
| 1. **The type of action:** polic* OR “prevention program*” OR strateg* OR program* OR practic* OR government*OR law |
| 1. **The design (reviews):** "data synthesis" OR "synthesis of data" OR "descriptive synthesis" OR "evidence synthesis" OR "synthesis of evidence" OR "synthesis of available evidence" OR "best-evidence synthesis" OR "framework synthesis" OR "interpretive synthesis" OR "knowledge synthesis" OR "synthes* of knowledge" OR "literature synthesis" OR "synthesis of literature" OR "synthesis of the literature" OR metasynthesis OR meta-synthesis OR "narrative synthesis" OR "qualitative synthesis" OR "quantitative synthesis" OR "realist synthesis" OR "research synthesis" OR "synthesis of research" OR "synthesized research" OR "review and synthesis" OR "synthesis of qualitative research" OR "thematic synthesis" OR "updated synthesis" OR "congruent synthesis" OR "critical interpretive synthesis" OR "framework synthesis" OR "meta synthesis" OR "qualitative evidence synthesis" OR "refutational synthesis" OR "critical analysis of the literature" OR metaanaly* OR meta-analy* OR "meta analy*" OR "Retrospective analysis" OR "Qualitative analysis" OR "Quantitative analysis" OR "systematic map*" OR "systematic narrative" OR "systematic overview*" OR "systematic review*" OR "systematically review*" OR "systematized review" OR "overview of systematic reviews" OR "systematic assessment" OR "systematic literature mapping" OR "bibliographic search" OR "database search" OR "search of database*"OR "electronic search" OR handsearch* OR "hand search*" OR "search by hand" OR "keyword search" OR "literature search" OR "search of literature" OR "search of the literature" OR "search term*" OR "systematic search" OR "comprehensive search" OR "manual search" OR "article reviews" OR "review of articles" OR "literature review" OR "review literature" OR "review of the literature" OR "overview* of reviews" OR "overview of systematic reviews" OR "qualitative review" OR "quantitative review" OR "realist review" OR "a review" OR "articles are reviewed" OR "authors review" OR "clinical review" OR "complete review" OR "comprehensive review" OR "conduct a review" OR "critical review" OR "critically review" OR "current review" OR "descriptive review" OR "epidemiological review" OR "evidence review" OR "review of the evidence" OR "evidence-based review" OR "integrat* review" OR "research review*" OR "international review" OR "is reviewed" OR "mapping review" OR meta-review OR "narrative review" OR "practitioner review" OR "rapid review" OR "rapid realist* review" OR "review all studies" OR "review and synthesis" OR "review of current evidence" OR "review of current research" OR "review of effectiveness" OR "review of the effectiveness" OR "review of reported cases" OR "review of research" OR "review of reviews" OR "selected for review" OR "selective review" OR "state-of-the-art review*" OR "structured review" OR "theory driven review" OR "scoping study" OR "meta-study" OR "literature-based study" OR "studies were identified" OR "compilation of the literature" OR "compilation of studies" OR "inclusion criteria" OR "exclusion criteria" OR metaregression OR "evidence map*" OR meta-evidence OR "systemic review" |
| 1. **the outcomes:** “physical activity” OR active OR exercise OR sedentary OR sport OR fitness OR sedentary OR “screen use” OR diet OR nutrition OR fat OR snack OR fruit OR vegetable OR fiber OR fibre OR soda OR meal OR food OR “energy intake” OR calorie* OR obes* OR eat* |

Note: The keywords were selected based on previous reviews addressing similar issues (e.g., [1-5]). Applied limiters in published reviews: peer-reviewed publications of reviews applying systematic search of original research (including systematic, scoping, realist reviews, meta-analyses), academic journals, English language only. Applied limiters in stakeholders’ databases: documents approved/published by the respective organization, English language only.

References:

1. Hennessy EA, Johnson BT, Keenan C. Best Practice Guidelines and Essential Methodological Steps to Conduct Rigorous and Systematic Meta-Reviews. Appl Psychol Health Well-Being. 2019;11:353–81. https://doi.org/10.1111/aphw.12169

2. Horodyska K, Luszczynska A, Hayes CB, O’Shea MP, Langøien LJ, Roos G, et al. Implementation conditions for diet and physical activity interventions and policies: an umbrella review. BMC Public Health. 2015;15:1250. https://doi.org/10.1186/s12889-015-2585-5

3. Proctor E, Silmere H, Raghavan R, Hovmand P, Aarons G, Bunger A, et al. Outcomes for implementation research: Conceptual distinctions, measurement challenges, and research agenda. Adm Policy Ment Health. 2011;38(2):65–76. https://doi.org/10.1007/s10488-010-0319-7

4. Seward K, Finch M, Yoong SL, Wyse R, Jones J, Grady A, et al. Factors that influence the implementation of dietary guidelines regarding food provision in centre based childcare services: A systematic review. Prev Med. 2017;105:197–205. https://doi.org/10.1016/j.ypmed.2017.09.024

5. Wolfenden L, Jones J, Williams CM, Finch M, Wyse RJ, Kingsland M, et al. Strategies to improve the implementation of healthy eating, physical activity and obesity prevention policies, practices or programmes within childcare services. Cochrane Database Syst. Rev. 2016; 10:CD011779. https://doi.org/10.1002/14651858.cd012439.pub2

Supplementary Table S2

Description of analyzed material in reviews and stakeholder documents

|  | **Reviews** | **Stakeholder documents** |
| --- | --- | --- |
| Number of included documents | 25 | 17 |
| Total number of original studies | 747 | Not available |
| **Type of action** | both policies and interventions | both policies and interventions |
| **Analyzed behaviors** | 1. dietary behaviors only (*k* = 8; 32%); 2. physical activity only (*k* = 5; 20%); 3. both, physical activity and sedentary behaviors (*k* = 2; 8%); 4. diet behaviors and physical activity *(*k = 6, 24%); 5. diet behaviors, physical activity, and other behaviors (*k* = 4; 16% [e.g., smoking cessations, alcohol use, drug use, oral health, relaxation at work, various social and mental health issues such as violence or bullying, eating disorders or well-being]). | 1. diet behaviors only (*k* = 4; 23%); 2. physical activity only (*k* = 2; 12%); 3. diet, physical activity, sedentary behaviors and other behaviors (*k* = 11; 65%). |
| **Populations analyzed** | 1. general populations of adults (*k* = 10; 40%); 2. employees (*k* = 4; 16%); 3. children and adolescents (*k* = 11; 44%). | 1. general population (*k* = 7, 41.2%;); 2. clinical populations (e.g., obese children, adolescents, adults or population at high risk of developing diabetes combined with or people with chronic diseases e.g., heart disease; *k* = 2, 11.8%); 3. specific communities (e.g., local communities; k = 2, 11.82%); 4. population of pregnant and postpartum women at risk for obesity (*k* = 2, 11.8%); 5. overweight and obese children, adolescents, and adults (*k* = 2, 11.8%); 6. employees (*k* = 1; 5.9%), 7. vulnerable population (e.g., children of low-income families, pregnant women, older adults with disabilities and age related illness, prisoners (*k* = 1; 5.9%). |
| **Policy implementation settings** | 1. school settings (*k* = 10, 40%); 2. mixed settings (*k* = 6; 24%); 3. workplace settings (*k* = 4; 16%); 4. food retail settings (*k* = 2; 8%); 5. rural community settings; urban planning and housing environment and transportation (*k* = 1; 4%); 6. elite sport settings and non-elite sport or physical activity facilities (*k* = 1; 4%). | 1. mixed settings (*k* = 9; 53%); 2. communities or community and health care settings (*k* = 3; 17.7%); 3. clinical practice settings (*k* = 2; 11.8%); 4. health, social services, educational, and other settings involved in prevention of overweight among pregnant and postpartum women and educating on infant feeding (*k* = 2; 11.8%); 5. workplace settings only (*k* = 1; 5.9%). |
| **Countries where original studies were conducted** | 1. In *k* = 16 out of 25 (64%) reviews, the majority of included studies were conducted in countries with very high Human Development Index (HDI), such as the USA, Canada, Australia and at least one of European countries with high HDI (e.g. United Kingdom, Netherlands, Finland, Norway, Germany); 2. *k* = 2 out of 25 reviews (8%) analyzed studies conducted only in USA and Canada); *k* = 1 out of 25, (4%) in USA only; *k* = 1 out of 25, (4%) – in Canada only; 3. One review included studies conducted in European countries only (*k* = 1 out of 25 (4%), however did not provided details regarding particular countries; 4. n = 1 review (4%) analyzed studies conducted in Latin American only (such as Argentina, Brazil, Chile, Mexico); 5. Three reviews did not provide detailed information regarding the country/region of the included in the review studies (*k* = 3 out of 25, 12%); 6. The majority of the research was done in USA (n = 288 studies), followed by general category of European countries (such as United Kingdom, Netherlands, Finland, Norway, Germany; *k* = 44), Canada (*k* = 63), Australia (*k* = 52), and broad category of other countries (such as Latin American countries, e.g., Argentina, Brazil, Chile, Mexico; New Zealand, China, Japan, Israel, South Africa, Pakistan; *k* = 53). |  |

Supplementary Table S3

Coding for key constructs and coding principles for the CFIR

| **Key constructs coded** | **Definitions and operationalizations applied** | |
| --- | --- | --- |
| **Policy** | Reviews and stakeholder documents were coded as referring to policy if any of original studies included into the respective review/document addressed actions aiming at promotion of healthy diet and active lifestyle, that are developed and implemented (or enforced) with regional, national, or international public agencies (e.g. national government or local authorities) participating in respective processes. | |
| **Implementation** | Reviews and stakeholder documents were coded as referring to implementation determinants if they addressed barriers and facilitators for policy implementation process, strategies, or context. | |
| **Healthy diet** | Reviews and stakeholder documents were coded as referring to healthy diet, if original studies addressed policies for food composition, food labelling, health nutrition promotion, food provision, food retail, food prices, or food trade and investment [1]. | |
| **Physical activity, sedentary behavior** | Reviews and stakeholder documents were coded as referring to an increase of PA or a reduction of SB if they targeted respective behaviors across sectors such healthcare, sport/recreation, education, transport, environment, urban design or urban planning, etc. [2]. | |
| **Note to documents addressing health diet, PA, SB and other behaviors** | Besides reviews and stakeholder documents targeting one specific behavior (e.g. healthy nutrition), several included documents analyzed policies addressing various target behaviors. These documents were grouped into documents addressing: (1) policies aiming at healthy diet, improvement of PA and SB reduction; (2) policies aiming at healthy diet, PA increase, SB reduction and other behaviors (e.g., tobacco use, alcohol use). | |
| **School setting** | Reviews and stakeholder documents were coded as referring to school setting if the original studies referred to education-related setting, including preschools, educational day care centers for young children, primary, secondary, and high schools; reviews and stakeholder documents were coded as referring to workplace if the policies analyzed in the original studies targeted populations of employees or managers at workplace settings. | |
| **Coding principles for the CFIR framework: specific cases** | | **Examples of coding** |
| Each time a determinant is considered to refer to an organizational level it was coded within inner setting domain of CFIR. | | n/a |
| In case a determinant was difficult to categorized a fallowing procedure was applied: In case a particular determinant (barrier or facilitator) description considered an individual characteristics/properties/attitudes/ perceptions/beliefs etc. it was coded within CFIR *characteristics of individuals* domain. | | *negative student outlook on physical activity* |
| In case a description of determinant was too broad and did not mention any individuals’ characteristics it was considered as referring to organizational level and coded within inner setting level. | | *diminishing priority [compared to other subjects]* |
| In cases: a determinant was referring to leadership, however did not mentioned any policy implementation stages/process it was coded within *readiness for implementation* domain (leadership engagement construct), rather than domain *process* (construct: opinion leaders). | | *leadership and support from staff, administration and other school champions* |
| When a determinant consisted of two disjunctive aspects/characteristics/facets it was separated and assigned to two different domains/constructs. | | *“lack of monitoring/accountability”* was divided and assigned to two different constructs within *process* domain – lack of monitoring to the category “*reflecting and evaluating”*, whereas *accountability* was assigned to the category “*opinion leaders”* |
| In case a determinant was referring to any characteristic of physical environment or infrastructure appearing in the organizational settings it was coded within *inner* setting domain (*structural characteristics* construct). | | *classroom influences, i.e., noise, disruption* |
| If it was unclear whether a determinant was considering an individual or organizational level or a process of implementation, it was not classified to any of the domains. | | *putting daily physical activity in schedule* |
| In case a barrier or facilitator was referring to any type of *skills* (e.g., *lack of skills and experience in physical activity delivery*), it was considered as related to individual level, rather than to organizational level. However, any type determinant described as *training* even when it was not mentioned as applying to individual or organizational level, it was coded as organizational and included within *inner setting* domain (e.g., *lack of training*, *training opportunities*). | | *lack of skills and experience in physical activity delivery*  *lack of training*, *training opportunities* |
| Determinants related to goals/demands where considered to apply to organizational level (rather than individual) and coded within *inner setting* domain and *compatibility* construct (e.g., barrier: *competing academic curricular demands*). | | *competing academic curricular demands* |
| Determinants referring to structural characteristics of outer settings, such as *inclement weather* were excluded from coding since the CFIR does not specify/include such construct. | | *inclement weather* |
| Determinants referring to *networking* described as *the nature and quality of social networks and the nature and quality of formal and informal communications within an organization*, were coded within *inner setting* domain (*networks and communications* construct). On the other hand, determinants related to *networking* depicted as *the degree to which an organization is networked with other external organizations* it was coded within *outer setting* domain (*cosmopolitanism* construct). | | *the quality of social networks*  *networking with other organizations* |
| If it was unclear whether the description of networking refers to outer or inner setting, it was excluded from the coding. | | *networking* |

Note: CFIR - the consolidated framework for implementation research; PA - physical activity; SB - sedentary behavior.

References:

1. Vandevijvere S, Mackay S, D’Souza E, Swinburn B. The first INFORMAS national food environments and policies survey in New Zealand national food environments and policies survey in New Zealand: A blueprint country profile for measuring progress on creating healthy food environments. Obes Rev. 2019;20:141–60. https://doi.org/10.1111/obr.12850

2. World Health Organization. Health-enhancing physical activity (HEPA) policy audit tool (PAT). https://www.euro.who.int/__data/assets/pdf_file/0010/286795/Health-enhancing_physical_activityHEPApolicy_audit_toolPATVersion_2.pdf (2015). Accessed 8 Oct 2020.

Supplementary Table S4. Barriers and facilitators for policy implementation: evidence accumulated in systematic reviews coded according to the CFIR

| **Policy implementation determinants: levels and categories** | **1** | **2** | **3** | **4** | **5** | **6** | **7** | **8** | **9** | **10** | **11** | **12** | **13** | **14** | **15** | **16** | **17** | **18** | **19** | **20** | **21** | **22** | **23** | **24** | **25^*^** | **SUM** | **≥ 50% and ≥ 60% reviews^i^** |
| --- | --- | --- | --- | --- | --- | --- | --- | --- | --- | --- | --- | --- | --- | --- | --- | --- | --- | --- | --- | --- | --- | --- | --- | --- | --- | --- | --- |
| Behavior | DIET | | | | | | | | PA | | | | | PA, SB | | PA, DIET | | | | | | PA, DIET, OTHER BEHAVIORS | | | |  | |
| **Policy characteristics** | | | | | | | | | | | | | | | | | | | | | | | | | | | |
| Intervention source |  |  |  |  |  |  |  |  |  |  |  |  |  |  |  |  |  |  | + |  |  |  |  |  |  | **1** |  |
| Evidence strengths |  | **+** |  | **+** | **+** |  |  |  |  |  |  |  |  |  |  |  |  |  | **+** |  |  | + | + |  |  | **6** |  |
| Relative advantage |  |  |  |  | **+** |  |  |  | **+** |  |  |  |  | **+** |  | **+** |  |  | **+** |  |  | + | + |  |  | **7** |  |
| Adaptability |  |  | **+** |  |  |  |  |  |  |  |  |  |  | **+** |  | **+** |  |  | **+** |  |  | + | + |  |  | **6** |  |
| Triability |  |  |  |  |  |  |  |  |  |  |  |  |  |  |  |  |  |  |  |  |  | + |  |  |  | **1** |  |
| Complexity |  |  | **+** | **+** |  |  | **+** | **+** | **+** | **+** | **+** | **+** | **+** | **+** | **+** | **+** |  |  | **+** |  |  | + |  |  |  | **14** | **✓** |
| Quality (design, package) |  | **+** | **+** |  |  |  | **+** |  |  |  |  |  |  | **+** |  |  |  |  | **+** |  |  | + |  |  | + | **7** |  |
| Cost |  |  | **+** | **+** | **+** | **+** | **+** | **+** | **+** | **+** | **+** | **+** |  | **+** | **+** | **+** | **+** |  | **+** |  |  | + | + | + | + | **19** | **✓✓** |
| **Outer setting** | | | | | | | | | | | | | | | | | | | | | | | | | | | |
| Target groups’ needs and resources |  |  | **+** |  |  | **+** |  |  | **+** | **+** |  |  | **+** | **+** |  | **+** | **+** |  | **+** |  |  | + | + |  | + | **12** |  |
| Networking with other organizations |  | **+** | **+** |  | **+** | **+** | **+** | **+** |  |  | **+** | **+** |  | **+** | **+** | **+** |  | **+** | **+** |  |  | + | + |  | + | **16** | **✓✓** |
| Peer pressure |  | **+** |  |  |  | **+** |  |  |  |  |  | **+** |  |  |  |  |  |  | **+** |  |  |  |  |  |  | **4** |  |
| External policies |  | **+** | **+** | **+** | **+** | **+** |  | **+** | **+** |  | **+** | **+** | **+** | **+** |  | **+** |  |  | **+** |  |  | + | + |  | + | **16** | **✓✓** |
| **Inner setting** | | | | | | | | | | | | | | | | | | | | | | | | | | | |
| Structural character |  |  | **+** | **+** |  | **+** | **+** | **+** | **+** | **+** | **+** |  | **+** | **+** | **+** | **+** | **+** | **+** | **+** |  |  | + |  |  | + | **17** | **✓✓** |
| Networks, communication |  |  | **+** |  |  |  | **+** | **+** |  |  | **+** |  |  | **+** | **+** | **+** |  |  | **+** |  |  | + | + |  |  | **10** |  |
| Culture, norms, values |  | **+** | **+** | **+** | **+** | **+** | **+** | **+** | **+** |  | **+** |  |  | **+** |  | **+** | **+** |  | **+** |  |  | + | + |  | + | **16** | **✓✓** |
| Implementation climate | + | **+** | **+** | **+** |  | **+** | **+** | **+** | **+** |  |  | **+** | **+** | **+** | **+** | **+** | **+** |  | **+** | **+** |  | + | + | + | + | **20** | **✓✓** |
| Readiness for implementation | + | **+** | **+** | **+** |  | **+** | **+** | **+** |  |  |  |  | **+** | **+** | **+** | **+** | **+** | **+** | **+** | **+** |  | + |  | + | + | **18** | **✓✓** |
| **Characteristics of individuals** | | | | | | | | | | | | | | | | | | | | | | | | | | | |
| Knowledge, beliefs | + | **+** | **+** | **+** | **+** | **+** | **+** |  | **+** | **+** | **+** | **+** | **+** | **+** | **+** | **+** | **+** | **+** | **+** |  |  | + | + |  | + | **21** | **✓✓** |
| Self-efficacy | + |  |  |  |  | **+** |  |  | **+** | **+** |  |  |  | **+** |  | **+** |  | **+** | **+** |  |  | + | + |  | + | **11** |  |
| Stage of change/ enthusiasm | + | **+** |  |  |  | **+** |  | **+** | **+** | **+** |  | **+** | **+** | **+** |  |  |  |  | **+** |  |  | + | + | + | + | **14** | **✓** |
| Identification with organization |  | **+** |  |  |  |  |  |  |  |  |  |  |  |  |  |  |  |  |  |  |  |  | + |  |  | **2** |  |
| Motivation, values, capacity |  | **+** | **+** | **+** | **+** | **+** |  |  | **+** | **+** |  | **+** | **+** | **+** | **+** | **+** | **+** | **+** | **+** |  |  | + | + |  | + | **18** | **✓✓** |
| **Process of implementation** | | | | | | | | | | | | | | | | | | | | | | | | | | | |
| Planning |  |  | **+** |  |  |  |  |  |  |  |  |  |  | **+** | **+** |  |  |  | **+** |  |  | + | + |  |  | **6** |  |
| Engaging leaders, external agents, champions | + | **+** |  |  | **+** | **+** |  | **+** | **+** |  |  |  | **+** | **+** | **+** | **+** |  | **+** | **+** | **+** |  | + | + |  | + | **16** | **✓✓** |
| Executing plans |  |  |  |  |  |  |  |  |  |  |  |  |  | **+** |  |  |  |  | **+** |  |  | + | + |  |  | **4** |  |
| Reflecting and evaluating |  | **+** |  | **+** |  |  |  | **+** |  |  |  |  | **+** | **+** | **+** | **+** | **+** |  | **+** | **+** |  |  |  |  |  | **10** |  |

Note: PA - physical activity; SB - sedentary behavior. ^*^ Numbers of the reviews (1-25) reflects the numbers of the reviews in the Supplement 1 (see also the list below). + - the reviews/stakeholder documents, that provided an explicit reference for a significant role/importance of a respective CFIR-related implementation determinant.

^i^ ≥ 50% (≥13 reviews) indicating preliminary support for determinant is marked with 🗸; ≥ 60.0% (≥15 reviews) indicating strong support for determinant is marked with 🗸🗸.

Reference list of systematic reviews (1-25) included in the Table S4

1. Seward K, Finch M, Yoong SL, Wyse R, Jones J, Grady A, et al. Factors that influence the implementation of dietary guidelines regarding food provision in centre based childcare services: A systematic review. Prev Med. 2017;105:197–205. https://doi.org/10.1016/j.ypmed.2017.09.024

2. Cullerton K, Donnet T, Lee A, Gallegos D. Playing the policy game: a review of the barriers to and enablers of nutrition policy change. Public Health Nutr. 2016;19:2643–53. https://doi.org/10.1017/S1368980016000677

3. Middel CNH, Schuitmaker-Warnaar TJ, Mackenbach JD, Broerse JEW. Systematic review: a systems innovation perspective on barriers and facilitators for the implementation of healthy food-store interventions. Int J Behav Nutr Phys Act. 2019;16:108. https://doi.org/10.1186/s12966-019-0867-5

4. Bergallo P, Castagnari V, Fernández A, Mejía R. Regulatory initiatives to reduce sugar-sweetened beverages (SSBs) in Latin America. PLoS One. 2018;13:e0205694. https://doi.org/10.1371/journal.pone.0205694

5. Eykelenboom M, van Stralen MM, Olthof MR, Schoonmade LJ, Steenhuis IHM, Renders CM. Political and public acceptability of a sugar-sweetened beverages tax: a mixed-method systematic review and meta-analysis. Int J Behav Nutr Phys Act. 2019;16:1356. https://doi.org/10.1186/s12966-019-0843-0

6. Houghtaling B, Serrano EL, Kraak VI, Harden SM, Davis GC, Misyak SA. A systematic review of factors that influence food store owner and manager decision making and ability or willingness to use choice architecture and marketing mix strategies to encourage healthy consumer purchases in the United States, 2005–2017. Int J Behav Nutr Phys Act. 2019;16:5. https://doi.org/10.1186/s12966-019-0767-8

7. Wang D, Stewart D. The implementation and effectiveness of school-based nutrition promotion programmes using a health-promoting schools approach: a systematic review. Public Health Nutr. 2013;16:1082-100.  https://doi.org/10.1017/S1368980012003497

8. McIsaac J-LD, Spencer R, Chiasson K, Kontak J, Kirk SFL. Factors Influencing the Implementation of Nutrition Policies in Schools: A Scoping Review. Health Educ Behav. 2019;46:224–50. https://doi.org/10.1177/1090198118796891

9. Nathan N, Elton B, Babic M, McCarthy N, Sutherland R, Presseau J, et al. Barriers and facilitators to the implementation of physical activity policies in schools: A systematic review. Prev Med. 2018;107:45–53. https://doi.org/10.1016/j.ypmed.2017.11.012.

10. Rees R, Kavanagh J, Harden A, Shepherd J, Brunton G, Oliver S, Oakley A. Young people and physical activity: a systematic review matching their views to effective interventions. Health Educ Res. 2006;21:806-25. https://doi.org/10.1093/her/cyl120

11. Heath GW, Brownson RC, Kruger J, Miles R, Powell KE, Ramsey LT. Task Force on Community Preventive Services. The Effectiveness of Urban Design and Land Use and Transport Policies and Practices to Increase Physical Activity: A Systematic Review. J Phys Act Health. 2006;3:S55-S76. https://doi.org/10.1123/jpah.3.s1.s55

12. Leone L, Pesce C. From Delivery to Adoption of Physical Activity Guidelines: Realist Synthesis. Int J Environ Res Public Health. 2017;14:1193. https://doi.org/10.3390/ijerph14101193

13. Weatherson KA, Gainforth HL, Jung ME. A theoretical analysis of the barriers and facilitators to the implementation of school-based physical activity policies in Canada: a mixed methods scoping review. Implement Sci. 2017;12:36. https://doi.org/10.1186/s13012-017-0570-3

14. Cassar S, Salmon J, Timperio A, Naylor P-J, van Nassau F, Contardo Ayala AM, et al. Adoption, implementation and sustainability of school-based physical activity and sedentary behaviour interventions in real-world settings: a systematic review. Int J Behav Nutr Phys Act. 2019;16:120. https://doi.org/10.1186/s12966-019-0876-4

15. Umstattd Meyer MR, Perry CK, Sumrall JC, Patterson MS, Walsh SM, Clendennen SC, et al. Physical Activity-Related Policy and Environmental Strategies to Prevent Obesity in Rural Communities: A Systematic Review of the Literature, 2002-2013. Prev Chronic Dis. 2016;13:E03. https://doi.org/10.5888/pcd13.150406

16. Naylor P-J, Nettlefold L, Race D, Hoy C, Ashe MC, Wharf Higgins J, McKay HA. Implementation of school based physical activity interventions: A systematic review. J Prev Med. 2015;72:95-115. https://doi.org/10.1016/j.ypmed.2014.12.034

17. Dugdill L, Brettle A, Hulme C, Bartys S, Long A. A review of effectiveness of workplace health promotion interventions on physical activity and what works in motivating and changing employees health behaviour. Project Report. London, UK.: NICE. 2007. http://www.nice.org.uk/guidance/index.jsp?action=download&o=36393

18. Wolfenden L, Barnes C, Jones J, Finch M, Wyse RJ, Kingsland M, et al. Strategies to improve the implementation of healthy eating, physical activity and obesity prevention policies, practices or programmes within childcare services. Cochrane database Syst Rev. 2020;2:CD011779. https://doi.org/10.1002/14651858.cd011779.pub2

19. van der Kleij R, Coster N, Verbiest M, van Assema P, Paulussen T, Reis R, Crone M. Implementation of intersectoral community approaches targeting childhood obesity: a systematic review. Obes Rev. 2015;16:454-72. https://doi.org/10.1111/obr.12273

20. McFadyen T, Chai LK, Wyse R, Kingsland M, Yoong SL, Clinton-McHarg T, et al. Strategies to improve the implementation of policies, practices or programmes in sporting organisations targeting poor diet, physical inactivity, obesity, risky alcohol use or tobacco use: a systematic review. BMJ Open. 2018;8:e019151. https://doi.org/10.1136/bmjopen-2017-019151

21. Anderson LM, Quinn TA, Glanz K, Ramirez G, Kahwati LC, Johnson DB, et al. The effectiveness of worksite nutrition and physical activity interventions for controlling employee overweight and obesity: a systematic review. Am J Prev Med. 2009;37:340-57. https://doi.org/10.1016/j.amepre.2009.07.003

22. Wierenga D, Engbers LH, van Empelen P, Duijts S, Hildebrandt VH, van Mechelen W. What is actually measured in process evaluations for worksite health promotion programs: a systematic review. BMC public health. 2013;13:1190. https://doi.org/10.1186/1471-2458-13-1190

23. Durlak JA, DuPre EP. Implementation matters: a review of research on the influence of implementation on program outcomes and the factors affecting implementation. Am J Community Psychol. 2008;41:327-50. https://doi.org/10.1007/s10464-008-9165-0

24. Wolfenden L, Regan T, Williams CM, Wiggers J, Kingsland M, Milat A, et al. Strategies to improve the implementation of workplace-based policies or practices targeting tobacco, alcohol, diet, physical activity and obesity. Cochrane Database Syst Rev. 2016;34:175. https://doi.org/10.1002/14651858.cd012439.pub2

25. Herlitz L, MacIntyre H, Osborn T, Bonell C. The sustainability of public health interventions in schools: a systematic review. Implement Sci. 2020;15:4. https://doi.org/10.1186/s13012-019-0961-8

Supplementary Table S5. Barriers and facilitators for policy implementation: evidence accumulated in stakeholder documents coded according to the CFIR

| **Policy implementation determinants: levels and categories** | **1s** | **2s** | **3s** | **4s** | **5s** | **6s** | **7s** | **8s** | **9s** | **10s** | **11s** | **12s** | **13s** | **14s** | **15s** | **16s** | **17s^*^** | **SUM** | **≥50% and ≥60% documents^i^** |
| --- | --- | --- | --- | --- | --- | --- | --- | --- | --- | --- | --- | --- | --- | --- | --- | --- | --- | --- | --- |
| Type of analyzed behavior | DIET | | | | PA | | DIET, PA, SB AND OTHER BEHAVIORS | | | | | | | | | | |  |  |
| **Policy characteristics** | | | | | | | | | | | | | | | | | | | |
| Intervention source |  |  |  |  |  |  |  |  |  |  |  |  |  |  |  |  |  | **-** |  |
| Evidence strengths | + |  |  | + |  |  |  | + | + | + | + |  | + | + |  |  |  | **8** |  |
| Relative advantage |  |  |  |  |  |  |  |  |  |  |  |  | + |  |  | + |  | **2** |  |
| Adaptability |  |  |  |  |  |  | + |  |  | + |  |  |  |  | + | + |  | **4** |  |
| Triability |  |  |  |  |  |  |  |  |  |  |  |  |  |  |  |  |  | **-** |  |
| Complexity |  |  |  |  |  |  |  |  |  |  |  |  |  |  |  |  |  | **-** |  |
| Quality (design, package) |  |  |  |  |  |  |  |  |  |  |  |  |  |  |  |  |  | **-** |  |
| Cost | + | + | + | + |  |  |  | + | + | + | + | + | + | + | + | + |  | **13** | **✓✓** |
| **Outer setting** | | | | | | | | | | | | | | | | | | | |
| Target groups’ needs and resources | + |  |  | + |  |  | + | + | + | + | + | + | + | + |  | + |  | **11** | **✓✓** |
| Networking with other organizations | + | + |  | + |  | + | + | + | + | + |  | + | + | + | + | + |  | **13** | **✓✓** |
| Peer pressure |  |  |  |  |  |  |  |  |  |  |  |  |  |  |  |  |  | **-** |  |
| External policies | + | + | + | + |  |  | + | + | + | + | + | + | + | + | + | + |  | **14** | **✓✓** |
| **Inner setting** | | | | | | | | | | | | | | | | | | | |
| Structural character | + |  | + |  | + |  | + | + | + | + |  | + |  | + |  | + | + | **11** | **✓✓** |
| Networks, communication | + | + |  | + |  | + | + | + |  | + |  | + | + | + | + | + |  | **12** | **✓✓** |
| Culture, norms, values |  |  |  |  |  |  | + | + | + | + | + |  | + | + | + | + |  | **9** | **✓** |
| Implementation climate |  |  |  | + | + | + | + | + |  | + |  | + | + | + | + | + |  | **11** | **✓✓** |
| Readiness for implementation |  | + |  |  |  | + | + | + | + | + | + | + | + | + | + | + | + | **13** | **✓✓** |
| **Characteristics of individuals** | | | | | | | | | | | | | | | | | | | |
| Knowledge, beliefs | + |  | + | + |  | + | + | + | + |  | + |  |  | + |  | + | + | **11** | **✓✓** |
| Self-efficacy |  |  |  |  |  |  |  |  |  |  |  |  |  |  |  |  |  | **-** |  |
| Stage of change/ enthusiasm |  |  |  |  |  | + |  |  |  |  |  |  |  |  |  |  | + | **2** |  |
| Identification with organization |  |  |  |  |  |  |  |  |  |  |  |  |  |  |  |  |  | **-** |  |
| Motivation, values, capacity |  |  |  |  |  | + | + | + |  | + | + |  |  |  |  | + | + | **7** |  |
| **Process of implementation** | | | | | | | | | | | | | | | | | | | |
| Planning |  | + |  |  |  | + |  |  | + |  |  | + |  |  |  | + |  | **5** |  |
| Engaging leaders, external agents, champions |  |  |  |  |  | + |  | + |  |  |  | + | + | + | + | + |  | **7** |  |
| Executing plans |  | + |  |  |  |  |  |  |  |  |  |  |  |  |  |  |  | **1** |  |
| Reflecting and evaluating | + | + |  |  |  | + | + |  | + | + |  | + | + | + | + | + |  | **11** | **✓✓** |

Note: PA - physical activity; SB - sedentary behavior. ^*^ Numbers of the stakeholder documents (1s-17s) reflects the numbers of the documents in the Supplement 1 (see also the list below). + - the reviews/stakeholder documents, that provided an explicit reference for a significant role/importance of a respective CFIR-related implementation determinant. ^i^ ≥ 50% (≥ 9 stakeholder documents) indicating preliminary support for determinant is marked with 🗸; ≥ 60.0% (≥ stakeholder 11 documents) indicating strong support for determinant is marked with 🗸🗸.

Reference list of stakeholder documents (1s-17s) included into Table S5

1. National Health and Medical Research Council. Australian dietary guidelines. Canberra, A.C.T.: National Health and Medical Research Council; 2013.

2. European Commission. Inspiring the shift from nutrition policy to implementation: How existing data can support nutrition decision-making in Guatemala. Brussels: Directorate General International Cooperation and Development; 2019. http://www.nipn-nutrition-platforms.org/IMG/pdf/nipn_guatemala_case_study_-_brief_-_july_2019.pdf. Accessed 8 Oct 2020.

3. Adler NE, Cutler DM, Fielding JE, Galea S, Glymour MM, Koh HK, et al. Addressing Social Determinants of Health and Health Disparities: A Vital Direction for Health and Health Care. NAM Perspectives. 2016;6. . https://doi.org/10.31478/201609t

4. Heymsfield S, Aronne LJ, Eneli I, Kumar R, Michalsky M, Walker E, et al. Clinical Perspectives on Obesity Treatment: Challenges, Gaps, and Promising Opportunities. NAM Perspectives. 2018;8. https://doi.org/10.31478/201809b

5. National Institute for Health and Care Excellence (NICE). Physical activity in the workplace: Public health guideline [PH13]. UK: National Institute for Health and Care Excellence (NICE); 2008. https://www.nice.org.uk/guidance/ph13/resources/physical-activity-in-the-workplace-pdf-1996174861765. Accessed 8 Oct 2020

6. National Institute for Health and Care Excellence (NICE). Physical activity: walking and cycling: Public health guideline [PH41]. UK: National Institute for Health and Care Excellence (NICE); 2012. https://www.nice.org.uk/guidance/ph41/resources/physical-activity-walking-and-cycling-pdf-1996352901061. Accessed 8 Oct 2020.

7. National Health and Medical Research Council. Clinical practice guidelines for the management of overweight and obesity in adults, adolescents and children in Australia. Melbourne: National Health and Medical Research Council; 2013.

8. National Health and Medical Research Council. Cultural competency in health: A guide for policy, partnerships, and participation. Canberra, A.C.T.: National Health and Medical Research Council; 2006.

9. National Health and Medical Research Council. Infant feeding guidelines: Information for health workers. Canberra, A.C.T.: National Health and Medical Research Council; 2012.

10. Colagiuri S, Johnson G. Case for Action proposal: A Comprehensive Type 2 Diabetes Prevention Program. Canberra, A.C.T.: Submitted by the NHMRC Research Translation Faculty Diabetes Mellitus Steering Group; 2014.

11.Teede H, Harrison C, editors. Case for Action proposal: Obesity prevention through preventing excess weight gain during pregnancy and postpartum. Canberra, A.C.T.: Submitted by the NHMRC Research Translation Faculty Obesity Steering Group; 2014.

12. European Commission. Reviews of scientific evidence and policies on nutrition and physical activity. Objective area A2: effectiveness and efficiency of policies and interventions on diet and physical activity. Brussels: Publications Office of the European Union; 2018. https://data.europa.eu/doi/10.2875/337727. (2018). Accessed 12 Oct 2020.

13. Dietz WH, Brownson RC, Douglas CE, Dreyzehner JJ, Goetzel RZ, Gortmaker SL, et al. Chronic Disease Prevention: Tobacco, Physical Activity, and Nutrition for a Healthy Start: A Vital Direction for Health and Health Care. NAM Perspectives. 2016;6. https://doi.org/10.31478/201609j

14. Dietz WH, Belay B, Bradley D, Kahan S, Muth ND, Sanchez E, et al. A Model Framework That Integrates Community and Clinical Systems for the Prevention and Management of Obesity and Other Chronic Diseases. NAM Perspectives. 2017;7. https://doi.org/10.31478/201701b

15. Zellner S, Bowdish L. The ROI of Health and Well-Being: Business Investment in Healthier Communities. NAM Perspectives. 2017;17. https://nam.edu/wp-content/uploads/2017/11/The-ROI-of-Health-and-Well-Being.pdf. Accessed 28 Oct 2020

16. National Institute for Health and Care Excellence (NICE). Obesity: working with local communities: Public health guideline [PH42]. UK: National Institute for Health and Care Excellence (NICE); 2012. https://www.nice.org.uk/guidance/ph42. Accessed 28 Oct 2020.

17. National Institute for Health and Care Excellence (NICE). Obesity prevention: Clinical guideline [CG43]. UK: National Institute for Health and Care Excellence (NICE); 2006. https://www.nice.org.uk/guidance/cg43. Accessed 28 Oct 2020.
